# Maternal interleukin 6 in pregnancy is associated with *everyday, but not test-based* executive functioning in 10-year-old children

**DOI:** 10.1017/S0033291725000674

**Published:** 2025-04-11

**Authors:** Parisa Mohammadzadeh, Jens Richardt Møllegaard Jepsen, Cecilie K. Lemvigh, Julie B. Rosenberg, María Hernández-Lorca, Astrid Sevelsted, Rebecca Vinding, Nilo Vahman, David Horner, Mikkel E. Sørensen, Kristina Aagaard, Casper-Emil T. Pedersen, Susanne Brix, Birgitte Fagerlund, Ann-Marie M. Schoos, Jakob Stokholm, Bo Chawes, Christos Pantelis, Birte Y. Glenthøj, Klaus Bønnelykke, Bjørn H. Ebdrup

**Affiliations:** 1 Center for Neuropsychiatric Schizophrenia Research (CNSR) & Centre for Clinical Intervention and Neuropsychiatric Schizophrenia Research (CINS), Mental Health Centre Glostrup, University of Copenhagen, Glostrup, Denmark; 2 Copenhagen Prospective Studies on Asthma in Childhood (COPSAC), Herlev and Gentofte Hospital, University of Copenhagen, Gentofte, Denmark; 3 Faculty of Health and Medical Sciences, Department of Clinical Medicine, University of Copenhagen, Copenhagen, Denmark; 4 Child and Adolescent Mental Health Center, Copenhagen University Hospital – Mental Health Services CPH, Copenhagen, Denmark; 5 Department of Biotechnology and Biomedicine, Technical University of Denmark, Kgs. Lyngby, Denmark; 6 Department of Psychology, University of Copenhagen, Copenhagen, Denmark; 7 Department of Pediatrics, Slagelse Hospital, Slagelse, Denmark; 8 Section of Microbiology and Fermentation, Department of Food Science, University of Copenhagen, Frederiksberg, Denmark; 9 Melbourne Neuropsychiatry Centre, Department of Psychiatry, The University of Melbourne and Melbourne Health, Carlton South, VIC, Australia

**Keywords:** cognitive outcomes, executive functioning, interleukin 6, maternal inflammation, preventive strategies

## Abstract

**Background:**

Elevated maternal interleukin 6 (IL-6) during pregnancy has been associated with adverse fetal brain development and neurodevelopmental disorders, which often involve executive functioning (EF) impairments. However, the association between maternal IL-6 levels during pregnancy and EF remains largely unexplored.

**Methods:**

The COPSYCH study is based on the prospective COPSAC2010 birth cohort of 700 mother-child pairs, recruited during pregnancy. The children’s executive functioning was assessed at age 10 using: (i) the Behavior Rating Inventory of Executive Function, Second Edition (BRIEF-2) parental questionnaire, and (ii) a comprehensive neuropsychological test battery. Maternal blood levels of IL-6 and hs-CRP were measured at gestational week 24. Associations between IL-6 (main analysis) and hs-CRP (secondary analysis) and EF in children at age 10 were investigated with regression models with extensive confounder adjustment.

**Results:**

Six hundred and four children (86% of the cohort) completed the 10-year follow-up. Higher maternal IL-6 levels were significantly associated with less efficient parental-rated executive functioning in the children: BRIEF-2 Global Executive Composite score (p = 0.003), Behavior Regulation Index (p = 0.005), Emotion Regulation Index (p=0.04), and Cognitive Regulation Index (p=0.007). Interaction analysis with sex was significant (p-value=0.01) and exploratory analyses showed that IL-6 associations to BRIEF-2 were solely driven by boys. Associations between IL-6 and neuropsychological tests, as well as associations between hs-CRP and EF outcomes, were non-significant.

**Conclusion:**

IL-6 during pregnancy was associated with less efficient everyday EF in children at age 10. If replicated, preventive strategies targeting inflammation in pregnancy may ameliorate adverse cognitive outcomes in offspring.

## Background

Executive functioning (EF) refers to higher-level cognitive abilities that enable behavior in a goal-directed manner, allowing the individual to adapt to changing or unfamiliar circumstances. EF is primarily related to frontal lobe functions (Diamond, 2013; Friedman & Miyake, 2017). EF is a broad cognitive domain often clustered into three main functions: response inhibition, working memory updating, and mental flexibility which are characterized as shared, yet distinct functions Adaptive human behavior is widely dependent on EF, playing a vital role in daily life, academic achievement, and occupational success (Miller, Nevado-Montenegro, & Hinshaw, 2012; Pearson et al., 2016). Consequently, individuals who exhibit good performance in EF tend to demonstrate resilience towards adverse life events and generally experience a higher quality of life (Diamond, 2013; Masten & Tellegen, 2012; Miller et al., 2012). Conversely, individuals with impaired EF are more prone to risk-seeking behavior and externalizing problems (McKewen et al., 2019; Mullin, Perks, Haraden, Snyder, & Hankin, 2020). Lastly, impaired EF increases vulnerability to various manifestations of psychopathology, and impaired EF is common across most neuropsychiatric disorders, especially neurodevelopmental disorders (Diamond, 2013; Harden et al., 2020). Hence, impaired EF has been suggested as a transdiagnostic factor and thus an important endophenotypic trait (Zelazo, 2020).

Interleukin 6 (IL-6), traditionally associated with immune response, has also been shown to play a role in neurodevelopment, influencing neuronal proliferation, cellular differentiation, and neuronal cell survival (Hsiao & Patterson, 2011; Wu, Hsiao, Yan, Mazmanian, & Patterson, 2017). Rapidly produced in response to changes in homeostasis, such as infections and injuries, IL-6 is linked to the subsequent production of the acute-phase reactant C-reactive protein (CRP) (Del Giudice & Gangestad, 2018). Furthermore, IL-6 is considered a central mediator of the effects of the Maternal Immune Activation hypothesis, suggesting that intrauterine inflammatory disturbances can have adverse effects on fetal brain development (Smith, Li, Garbett, Mirnics, & Patterson, 2007; Wu et al., 2017). Ultimately, perturbations resulting in elevated maternal IL-6 levels during pregnancy are hypothesized to contribute to neurodevelopmental disorders, such as pervasive developmental disorders, hyperkinetic disorder, attention deficit disorder without hyperactivity, and schizophrenia (Allswede, Yolken, Buka, & Cannon, 2020; Gustafsson et al., 2020; Hsiao & Patterson, 2011; Wu et al., 2017).

Maternal IL-6 levels during pregnancy have also been linked to a negative impact on the intelligence of the offspring (Rasmussen et al., 2019). To our knowledge, only a limited number of studies have evaluated the association between higher inflammation during pregnancy and compromised EF in the offspring. One cohort study (N=84) reported that higher IL-6 during pregnancy was associated with reduced efficiency of impulse control (Graham et al., 2018) and working memory (Rudolph et al., 2018) in early life. Another study (N=40) found an indirect association between IL-6 and preschool EF through infant cognitive ability (Camerota et al., 2022). Dozmorov et al (N=246) did not find any significant association between maternal IL-6 and EF in the offspring (Dozmorov et al., 2018). Finally, a study by Morgan et al. (N=100) found higher maternal CRP levels during the third trimester were associated with poorer cognitive flexibility in offspring aged 4–6 years (Morgan et al., 2020).

In addition to exposure to inflammation during pregnancy, several environmental factors have been shown to influence EF. These include maternal and gestational factors, for example, birth weight, gestational age, preeclampsia, maternal age, smoking during pregnancy, maternal perinatal mental health, prenatal obesity, and socioeconomic status (Micalizzi & Knopik, 2018; Mina et al., 2017; Pearson et al., 2016; Rätsep et al., 2016; Sarsour et al., 2011; van Houdt, Oosterlaan, van Wassenaer-Leemhuis, van Kaam, & Aarnoudse-Moens, 2019).

Finally, EF etiology has a substantial genetic component, with twin studies reporting high heritability estimates when treated as a single common factor (Friedman & Miyake, 2017; Miguel, Meaney, & Silveira, 2023), the underlying genetic structure of EF is complex, and genome-wide association studies reveal a highly polygenic nature, with polygenic risk scores (PRS) currently explaining 10.4–18.9% of the variance (Hatoum et al., 2023; Miguel et al., 2023).

We used data from The COpenhagen Prospective Study on Neuro-PSYCHiatric Development (COPSYCH) study of the prospective Copenhagen Prospective Studies on Asthma in Childhood 2010 (COPSAC2010) birth cohort including 700 mother-child pairs representative of the general population. We investigated a association between maternal IL-6 levels (main analyses) and hs-CRP (secondary analyses) in pregnancy week 24 and every day- and performance-based EF in the offspring, at age 10. We hypothesized that a higher level of maternal inflammation would be associated with impaired EF in the child in middle childhood.

## Methods

### Study design and participants

The COPSYCH study included 10-year-old children from the deeply phenotyped prospective mother-child cohort, COPSAC2010, clinically assessed from January 2019 to December 2021 (Mohammadzadeh et al., 2022). All raters who administered the tests received extensive training and continuous supervision (*anonymized*). The COPSAC2010 cohort includes 700 unselected mother-child pairs where the children have undergone extensive phenotyping and biological sampling from 15 clinical visits from pregnancy to age 10 years (Bisgaard et al., 2013). The COPSAC2010 includes a double-blinded, randomized, and placebo-controlled study, in which pregnant women received either fish oil (2.4 g/day of n-3 long-chain polyunsaturated fatty acids or placebo, and/or high dose Vitamin D (70 microgram/day) or recommended standard dose Vitamin D (10 microgram/day) from 24 weeks of gestation until one week after birth. The primary outcome of the trial was asthma and persistent wheezing at age 3, and findings have been reported elsewhere (Bisgaard et al., 2016; Chawes et al., 2016).

For twin pairs, Twin B was excluded to ensure data independence, as five pairs of twins were included in COPSYCH. Additionally, children with a birth weight under 1,500 g or a gestational age under 28 weeks were excluded, though none of the participants in the COPSYCH visit met these criteria. Initial quality control was performed on the raw data, excluding unconfirmed extremely high values of biomarkers; one mother with an exceptionally high CRP level that could not be confirmed as an infections using a registered prescription of antibiotics (N=1). All IL-6 values were within expected ranges.

### Maternal inflammation

Blood levels of IL-6 and hs-CRP were measured at 24 weeks gestation for mothers. Serum IL-6 and hs-CRP were collected in EDTA tubes and blood samples were centrifuged and stored at −80° C. Concentrations were determined by a high-sensitivity electrochemiluminescence assay from MesoScale Discovery with a lower level of detection of 0.178 pg/mL for IL-6 and 0.007 ng/mL for hs-CRP. Samples were analyzed in duplicate and read using the Sector Image 2400A (Meso Scale Discovery, Gaithersburg, MD). Overall CV coefficients for IL6: 1.99% and for hs-CRP: 1.57%.

### Psychopathology

Neurodevelopment, as reflected by neurocognitive and psychopathological indices, was comprehensively assessed at the COPSYCH 10-year visit (Mohammadzadeh et al., 2022). Research diagnoses were based on the Kiddie Schedule for Affective Disorders and Schizophrenia Present and Lifetime Version (K-SADS-PL) as well as any other available clinical and anamnestic information (Kaufman et al., 1997). K-SADS-PL interviews were administered by trained personnel, video recorded, and supervised by a senior research psychologist with a specialty in child and adolescent psychiatry (*anonymized*). Consensus diagnoses or status as mentally healthy was established at a weekly diagnostic conference, followed by external validation from a clinical professor of child and adolescent psychiatry (*anonymized*). The distribution of diagnoses according to the ICD-10 Classification of Mental and Behavioral Disorders, 10th Revision (WHO, 1992) is provided in Table 1. We defined children with any neurodevelopmental disorder, according to ICD-10 codes, as follows: Pervasive Developmental Disorders (F84, F84.5, F84.8); Other Disorders of Psychological Development (F88); and Unspecified disorder of psychological development (F89), Hyperkinetic disorders (F90.0, F90.8); Attention deficit disorder without hyperactivity (F98.8); Chronic motor or vocal tics (F95.1); Tourette syndrome (F95.2).

**Table 1.** Baseline characteristics

Variable	COPSYCH participants	Range
*N*	604	
**Child characteristics**		
Age in years (SD)	10.29 (0.4)	[9.19–12.3]
Sex (Male): *N* (%)	313 (51.8)	
Race (non-white): *N* (%)	27 (4.5)	
**Birth/gestation**		
Gestational age in days: mean (SD)	279.30 (11.5)	[206–296]
Apgar (5 min): mean (SD)	9.93 (0.4)	[6–10]
Birth weight in kg: mean (SD)	3.55 (0.5)	[1.33–5.15]
Parity: mean (SD)	1.71 (0.8)	[1–6]
Delivery		
Normal: *N* (%)	474 (79.1)	
Planned section *N* (%)	52 (8.7)	
Acute section *N* (%)	73 (12.2)	
**Maternal characteristics**		
Age (at birth): years, mean (SD)	32.34 (4.3)	[19.06–48.3]
Mother BMI: mean (SD))	24.61 (4.43)	[15.55–45.81]
Smoking pr. Week: N (>1 unit) (%)	44 (7.3)	
Alcohol pr. Week: N (>1 unit) (%)	92 (15.3)	
Diabetes: N (%)	15 (2.5)	
Preeclampsia: N (%)	27 (4.5)	
Highest education at birth		
University: N (%)	174 (28.8)	
High school: N (%)	336 (55.6)	
**Paternal characteristics**		
Age at birth: years, mean (SD)	34.60 (5.22)	[23.05–53.07]
Highest education at birth		
University: N (%)	168 (28.5)	
High school: N (%)	241 (40.8)	
**Household income at birth, per annum (DKK)**		
<100,000	55 (9.1)	
100,000 – 150,000	139 (23.1)	
150,000 – 200,000	178 (29.5)	
200,000 – 250,000	139 (23.1)	
>250,000	92 (15.3)	
**Diagnoses (total examined N=593)**		
**ICD categorized**	**N (%)**	
F00–09 Organic, including symptomatic, mental disorder	–	
F10–19 Mental and behavioral disorders due to psychoactive substance use	–	
F20–29 Schizophrenia, schizotypal and delusional disorders	1 (0.2)	
F30–39 Mood [affective] disorders	–	
F40–49 Neurotic, stress–related and somatoform disorders	36 (7.2)	
F50–59 Behavioral syndromes associated with psychological disturbances and psychological factors	–	
F60–69 Disorders of adult personality and behavior	–	
F70–79 Mental retardation	–	
F80–89 Disorders of psychological development	33 (6.6)	
F90–98 Behavioral and emotional disorders with onset usually occurring in childhood and adolescence	138 (21)	
F99	–	
None	442 (74.5)	

Diagnoses: Frequency of diagnoses (Incl. phobia, enuresis, transient tic) found in the COPSYCH study (N=593, number of children that completed KSADS) according to classification. ‘–’ Indicates no diagnosis in that category. Idividuals presenting more than one condition are included in all the diagnostic categories. ICD-10 codes: codes for the diagnoses in the Classification of Mental and Behavioral disorders (ref); N (%): number of cases (percentage). Following diagnoses are included in the following categories: F20–29: Psychotic disorder (F28), F40–49: Anxiety disorder (phobia) (40.1, 40.2, 40.8), Obsessive-compulsive disorder (F42, F42.1, F42.2), Adjustment disorder (43.2), F80–89: Pervasive developmental disorder (F84, F84.5, F84.8), other neurodevelopmental disorders (F88 and F89), Hyperkinetic disorder (F90.0*, F90.8*), Conduct disorders (F91.1 F91.2 F91.3), Anxiety (F93.0, F93.2, F93.8), Transient tic disorder (F95.0), Chronic motor or vocal tics (F95.1), Tourette (F95.2), Non-organic nuresis, non-organic encopresis (F98.0, F98.1), Attention deficit disorder without hyperactivity (F98.8)

### Executive functioning

EF was assessed using two sources of data: an everyday executive functioning rating scale and performance-based EF test outcomes.

The parents evaluated the child’s everyday executive functioning behavior using the parental version of the Behavior Rating Inventory of Executive Function, second edition (BRIEF-2). BRIEF-2 is a 63-item questionnaire covering a broad range of executive functioning. BRIEF-2 has nine clinical subscales: Inhibit, Self-Monitor, Shift, Emotional Control, Initiate/Task Completion, Working Memory, Plan/Organize, Task-Monitor, Organization of Materials in their daily environment (G. A. Gioia, Isquith, Guy, & Kemworthy, 2015). All items are rated on a three-point Likert scale assessing frequency (‘*Never’, ‘Sometimes’, ‘Often’*) of the behavior during the last 6 months. The subscales can be combined into three index scores: Behavior Regulation Index, Emotion Regulation Index, and Cognitive Regulation Index. The sum of the three index scores represents the total score, denoted the Global Executive Composite score (Gioia et al., 2015).

**Figure 1.**
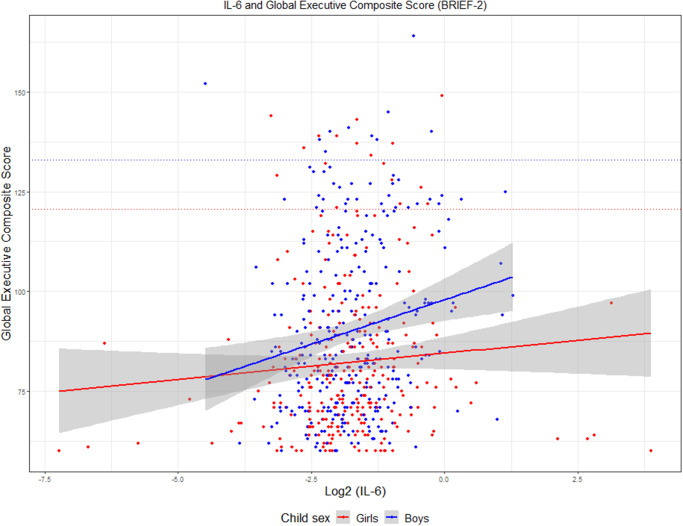
Association between maternal IL-6 level and Executive Composite Score, BRIEF 2, sex-stratified. Interleukin 6 in pregnancy week 24 (log2 transformed) and Global Executive Composite score, Behavior Rating Inventory of Executive Function, second edition, in offspring at age 10. Stratified by sex.

BRIEF-2 is validated in children aged 5–18 years, showing a high test-retest reliability, and standardized scores derived from normative samples are available (Davidson, Cherry, & Corkum, 2016; Gioia, Isquith, Guy, & Kenworthy, 2010). BRIEF-2 has previously been used in both neurotypical children and children with neurodevelopmental disorders (Thorup et al., 2015). For interpretation purposes, T scores (~2 SD) of 70 or higher are indicative of clinical levels of problems (Gioia et al., 2015). In the present cohort, this threshold corresponded to a Global Executive Composite raw score of 133.0 in boys and 122.7 in girls (Figure 1).

The neuropsychological battery included a broad range of well-validated tests (Andrikopoulos, 2021; De Luca et al., 2003; Delis, Kramer, Kaplan, & Holdnack, 2004). From the Cambridge Neuropsychological Test Automated Battery (CANTAB) (Fray & Robbins, 1996; Sahakian & Owen, 1992) we included following EF: Spatial Working Memory (SWM), testing spatial working memory and strategy formation; One-touch Stockings of Cambridge (OTS) testing planning; Spatial Span (SSP) testing spatial short-term memory, and Intra-Extra Dimensional Set Shifting (IED), testing mental flexibility (Table 2).

**Table 2.** Executive functioning outcomes

Parental assessment (questionnaire)			
Neurocognitive subdomain	Outcome variable	Median/mean [IQR]/(SD) (N = 604)	[Range]
*BRIEF–2 – (63 items)*			
Global executive composite¤		81 [70–97]	[60–164]
Behavior regulating index	- Impulse control, self-monitoring	15 [13–18]	[12–34]
Emotional regulating index	- Shift, emotional control	46 [18]	[32–94]
Cognitive regulating index	- Initiating, planning/organization, working memory and task monitoring	20 [8]	[16–48]

Child assessment (Tests)			
Neurocognitive subdomain	Outcome variable	Median [IQR]/mean (SD) (N = 604)	[Range]
*Flexibility/set shift*			
Intra-extra dimensional set shift (CANTAB)	-Extra-dimensional stage errors^a^	17 [6.5–27]	[0–34]
Trail-making test 4 – number-letter switching (D-KEFS)	-Time to complete in seconds	111.02 (38.1)	[49–240]
*Spatial working memory*			
Spatial span task (CANTAB)	- Forward span length^b^	5.96 (1.2)	[3–9]
Spatial working memory (CANTAB)	-Between errors	11 [4–17]	[0–32]
	-Strategy formation^c^	8 [7–9]	[2–13]
*Planning*			
One touch stockings of Cambridge (CANTAB)	-Problems solved in minimum moves	7.30 (1.9)	[2–12]
	-Mean initial thinking time (5 moves)	2882 [1823.7–4454.6]	[0.0–51763.3]
*Verbal working memory*			
Digit span (WISC-IV)	-Total number of correct forward and backward digit sequencing	14.13 (2.5)	[7–24]
Letter-number sequencing (WISC-IV)	-Total number of letter-number sequencing	17 [15–18]	[0–26]
*IQ*			
Estimated full-scale intelligence quotient^d^		102.7 (12.7)	[53–139]

WISC-IV, wechsler intelligence scale for children—fourth edition.

D-KEFS, Delis-Kaplan executive function system.

CANTAB, Cambridge neuropsychological test automated battery.

BRIEF-2, the behaviour rating inventory of executive function—second edition.

*¤Summary score calculated based on the three sub-indices Behavior regulating index, Cognitive regulating index, Emotion regulating index.*

^a^ *Extra-dimensional stage errors denote the number of errors made in the extra-dimensional stage of the task, where the child is required to make an extradimensional shift.*

^b^ *Span length denotes the longest sequence successfully recalled by the child (three attempts at each level).*

^c^ *Total errors comprise the number of times a box is selected that does not have a hidden target that must be found, and therefore, should not have been visited by the child.*

*Behavior Rating Inventory of Executive Function, Second Edition.*

^d^ *Estimated full-scale intelligence quotient, based on N=589 who underwent complete testing on monolingual children (bilingual did not perform the WISC vocabulary test, and therefore, they were not included).*

From the Wechsler Intelligence Scale for Children, Fourth Edition (WISC-IV) (David, 2003) we included the Digit Span, and Letter–Number Sequencing subtests to examine verbal working memory. Finally, Trail Making 4 from the Delis-Kaplan Executive Function System (D-KEFS), was used to measure mental flexibility (Delis, Kaplan, & Kramer, 2001). In addition to EF, we estimated the full-scale intelligence quotient (FSIQ) based on the following subtests from WISC-IV: Matrices, Vocabulary, Digit Span, Letter-Number Sequencing, Coding, and Symbol Search.

### Polygenic risk score for EF

In the absence of a maternal EF measure, we explored the genetic contribution to the children’s EF by generating a polygenic risk score (EF-PRS) for the children and mothers (EF-PRS), separately based on a genome-wide association study of executive function in 427,037 individuals from the UK Biobank (Hatoum et al., 2023).

For details on the construction of PRS, see Supplementary, Methods section.

### Weighing of outcome measures

EF rating scales and EF tests offer complementary information, but EF rating scales have higher ecological validity, and generally predict EF outcomes better than laboratory EF tests (Toplak, West, & Stanovich, 2013). Therefore, our primary outcome was the overall summary score from BRIEF-2, the Global Executive Composite score, and the three index scores: Behavior Regulation Index, Emotion Regulation Index, and Cognitive Regulation Index. Our secondary outcome was from a principal component analysis (PCA) of the neuropsychological EF test outcomes (EF-PCA). The main reasons for using PCA were to reduce variables for statistical efficiency and lower the risk of Type I errors. Additionally, PCA aligns with the theoretical framework of EF, considered ‘shared but distinct,’ allowing for collective examination (Friedman & Miyake, 2017). We included nine EF outcome variables from CANTAB, WISC-IV, and D-KEFS (Table 2) based on z-scored values from participants with complete EF test datasets. Four of the variables were inverted prior to making the PCA align so that higher z-scores consistently represented better performance (SWM strategy, SWM between errors, IED errors, and D-KEFS trail making).

Multiple testing correction using the Benjamini-Hochberg procedure was applied specifically to the four main outcomes from the BRIEF-2, with IL-6 as the primary determinant. The additional analyses were either secondary (hs-CRP) or exploratory and were not subjected to multiple testing corrections.

### Strengths and difficulties questionnaire (SDQ)

We included the SDQ as an outcome measure as a supplementary analysis, focusing on the externalizing subscale and the total difficulties score. The externalizing subscale consists of the behavioral and hyperactivity subscales (Goodman et al., 2010). For a description of the SDQ and the outcomes, see Supplementary Methods section.

### Statistical analysis

#### Main, secondary, and exploratory analysis

Maternal IL-6 and hs-CRP were log2 transformed in all analyses, for example, an increase in 1 is equivalent to a doubling of IL-6 and hs-CRP, respectively. The purpose was to depict the association across the whole range. Scores from BRIEF-2 are presented as raw scores. Missing data in the BRIEF-2 questionnaire were imputed by simple imputation; the first missing value, it was replaced by the value of 1, as indicated in the BRIEF-2 manual (Gioia et al., 2015). Subsequent missing values were imputed by the rounded mean of the remaining values within the same subscale.

All associations between the two maternal biomarkers and EF were investigated using linear regression, with IL-6-EF associations as the main analyses and hs-CRP-EF associations as secondary analyses. Associations were adjusted for: the child’s sex, gestational age, birth weight, parity, maternal and paternal age, pre-pregnancy maternal Body Mass Index (BMI), maternal education level, smoking during pregnancy, alcohol use during pregnancy, maternal diabetes during pregnancy, preeclampsia, antibiotic use during pregnancy, maternal EF-PRS, and household income. Furthermore, we adjusted for the RCT interventions (Vitamin D and fish oil).

In case the overall association between IL-6, respectively, hs-CRP and EF were significant we tested for interaction between the association and the following: sex, maternal education, household income, maternal pre-pregnancy BMI, birth weight, and any neurodevelopmental disorder present/absent, with Global Executive Composite score from BRIEF-2 as outcome. We evaluated whether the presence of neurodevelopmental disorders influenced an association by stratifying our analysis (exploratory). First, we excluded children with these diagnoses and performed the analysis. Then, we analyzed only children with neurodevelopmental disorders. We also conducted further analyses stratified by both sex and NDD status (results in Supplementary). All analyses were performed ‘unadjusted’, with age and sex being the only adjustments made, unless explicitly stated otherwise. This ‘unadjusted’ model included these two covariates given the significant influence of age at examination and sex on cognitive performance. In addition, we report estimates based on an ‘adjusted’ model, where the estimate was adjusted for both sex and age, along with all the covariates specified above. All results presented in the text are based on the main adjusted analyses unless otherwise specified. Results were reported as beta-coefficients with a 95% confidence interval [95%CI]. In all analyses, statistical significance was set as p-value<0.05 (two-sided). P-values for multiple BRIEF-2 outcomes were adjusted with the Benjamini–Hochberg correction method, and significance was determined based on these adjusted p-values.

Statistical analyses were performed using R statistical software (version R4.2.1, R Core Team (2022)).

#### Sensitivity analyses

The specificity of IL-6 and EF was investigated with an association analysis of estimated full-scale intelligence quotient and IL-6 levels. Furthermore, we performed correlational analyses using Spearman’s rank correlation coefficient, between EF-PCA outcomes and all nine clinical BRIEF-2 subscales. Finally, we repeated the models without PRS adjustment for all significant main analyses to assess potential genetic confounding, as PRS explains only a small fraction of the genetic contribution.

#### Supplementary analysis

For the SDQ supplementary analyses, we conducted both unadjusted and adjusted linear regression analyses to examine the associations between maternal inflammatory markers and SDQ outcomes. Additionally, we used Spearman’s correlation analysis to assess the bivariate relationships between the BRIEF-2 GEC and SDQ outcomes.

## Results

### Baseline

Six hundred and four children (86.3% of the original cohort) participated in the 10-year visit (see Table 1 for demographics). A total of five children met the exclusion criteria and this is a study of 599 children in total (85.6% of the original cohort). There were no significant differences in baseline characteristics between participants in the 10-year visit and non-participants (Supplementary Table S1). We had complete data for 577 children in the unadjusted analysis of maternal IL-6 and EF, and complete data including all covariates for 544 children (Figure S1).

Five hundred and fifty-one children with complete data (95.5% of 577) underwent psychopathological assessment, and 80 (13.9% out of 577) children fulfilled the criteria for at least one neurodevelopmental disorder (Table 1).

For baseline IL-6, hs-CRP, and BRIEF-2 data analyses, see Supplementary, Results section.

### Main analyses

#### Maternal pregnancy IL-6 levels and BRIEF-2 outcomes in offspring at 10 years of age

A total of 547 children (90.6%) had complete BRIEF-2 data. Nine (1.5%) did not fill out any items in the questionnaire (63/63 missing), 40 (6.6%) missed 1/63 values and were assigned the value ‘1’, and 8 (1.3%) had 2-11/63 missing values (Gioia et al., 2015).

**Figure 2.**
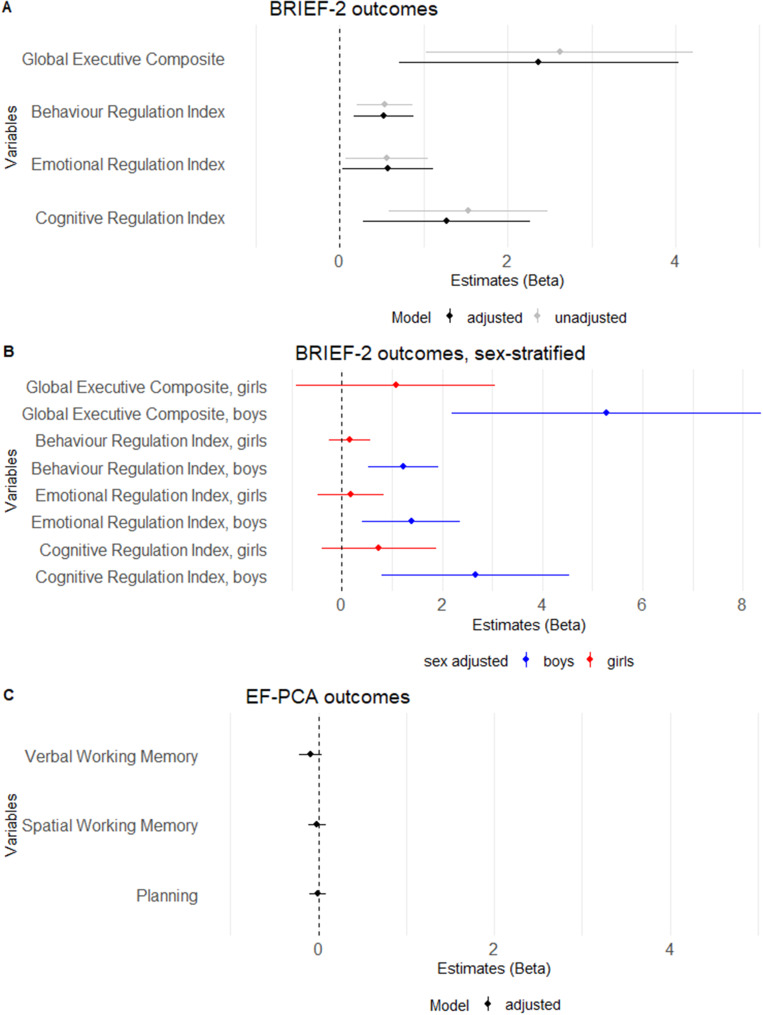
Forest plot showing an association between EF outcomes and maternal IL-6. Forest plot showing estimates and confidence intervals from linear regression analyses of maternal interleukin 6 level at pregnancy week 24 and A) Behavior Rating Inventory of Executive Function, second edition outcomes at age 10. (black=adjusted, grey=unadjusted); B) Behavior Rating Inventory of Executive Function, second edition outcomes at age 10, gender stratified (red=girls, blue=boys); and C) Executive Functioning-Principal Component Analysis outcomes at age 10, adjusted.

IL-6 in pregnancy was significantly associated with EF measured by the Global Executive Composite score (β[95%CI]=2.48 [0.80–4.16], p=0.004), indicating a 2.48 higher point score in EF problems per log2 increase in IL-6 (Figure 1). When subdivided into the three index scores we also observed significant associations with Behavior Regulation (β[95%CI]=0.52 [0.15–0.88] p=0.006), Emotion Regulation Index (0.58 [0.03–1.12] p=0.04), and Cognitive Regulation Index (1.39 [0.40–2.38], p=0.007) (Figure 2 and Supplementary Table S2).

#### Maternal pregnancy IL-6 levels and performance-based indices of executive functions

A total of 581 (96.19%) children who underwent cognitive examination had complete data. The PCA of the executive functioning test outcomes resulted in three components with an eigenvalue >1.00, and accounting for 55.17% of the variance. The components represented Verbal working memory with a 28.62% variance explained, Spatial working memory, 14.45% variance explained, and Planning, 12.10% variance explained (Figure S2).

We found a significant association between maternal IL-6 levels in pregnancy and the Verbal working memory component from the EF-PCA, but this finding became non-significant after adjusting for covariates (unadjusted: −0.16 [−0.29 to −0.04], p=0.01, adjusted: β[95%CI]=−0.09 [−0.22–0.04], p=0.17). We did not find a significant association between maternal IL-6 and the Spatial working memory or Planning components (Figure 2 and Supplementary Table S6).

Individual analyses for each EF test outcome can be found in Supplementary Table S7. All results were insignificant after adjustment.

### Secondary analyses

#### Maternal hs-CRP week 24 in pregnancy and EF outcomes

A significant association with BRIEF-2 outcomes was found in the unadjusted analyses but became insignificant after adjustment (Supplementary Table S3). No associations were found with EF-PCA (p-values > 0.12) or with the individual neuropsychological test outcomes (Supplementary Table S4).

### Explorative IL-6 analyses – analyses of effect modification

#### Effect modification by sex

**Figure 3.**
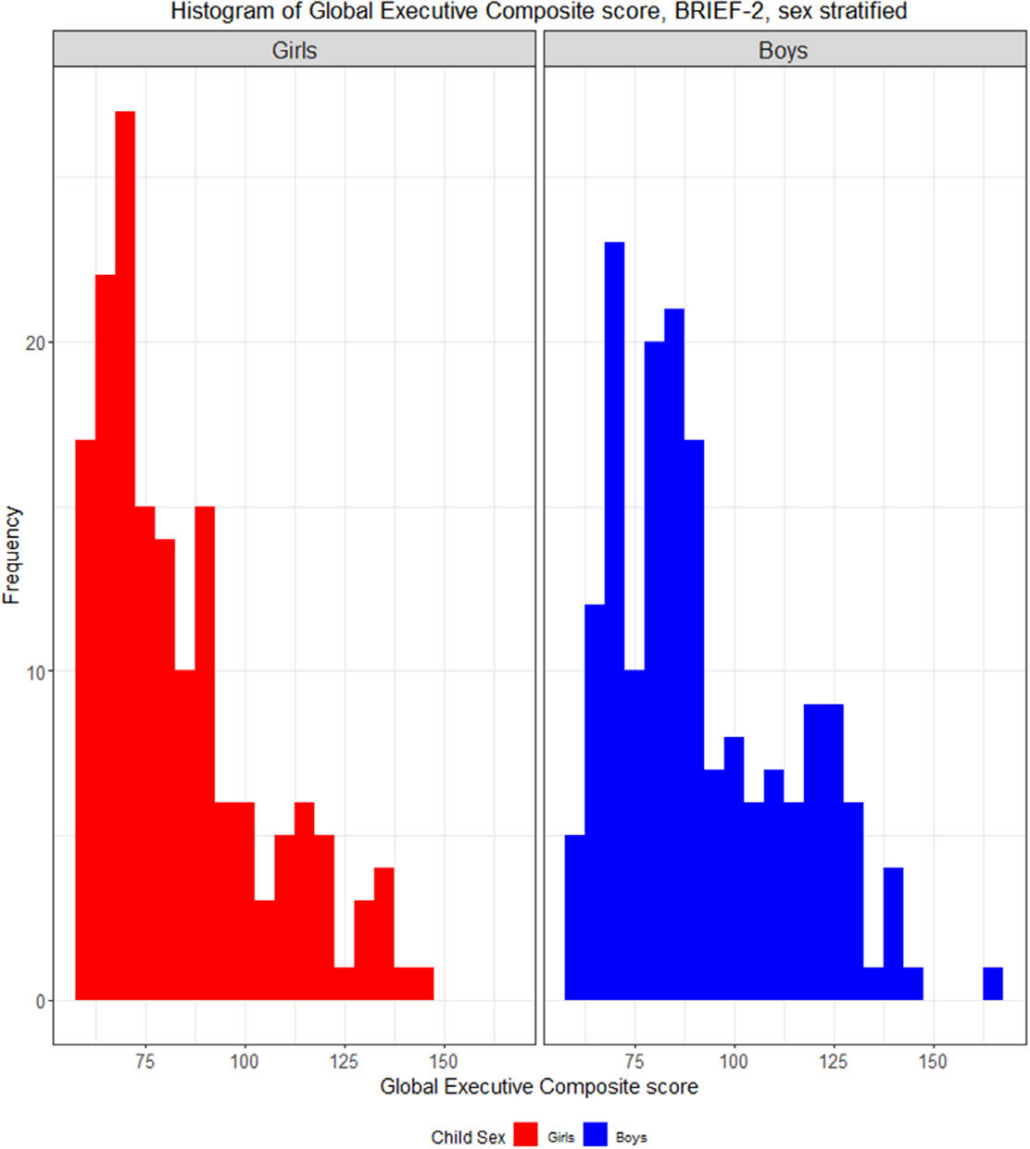
Distribution of the Global Executive Composite score from BRIEF-2, sex stratified. Histogram showing the Global Executive Composite scores (raw scores) from the Behavior Rating Inventory of Executive Function, second edition for girls and boys. Higher scores correspond to poorer performance.

Parents reported more frequent EF problems among boys than girls (Global Executive Composite girls: median [IQR]=77 [68.0–89.5] versus Global Executive Composite boys: median [IQR]=85 [72.0–104.25], p<0.001) (Figure 3). There was a significant interaction between maternal IL-6 levels in pregnancy and the child’s sex in relation to EF (p=0.01). Sex-stratified analyses showed that the association between the Global Executive Composite score and maternal IL-6 in pregnancy was only significant in boys (Global Executive Composite, males: β[95%CI]=5.28 [2.19–8.37], p<0.001; Global Executive Composite score, girls, 1.08 [−0.91–3.06], p=0.29). Behavior Regulation Index, Emotion Regulation Index, and Cognitive Regulation Index also exhibited significant associations with the maternal IL-6 level in pregnancy week 24 only in boys (Figure 2 and Supplementary Table S2).

#### Effect modification by neurodevelopmental disorders

Analyses evaluating the children according to the absence or presence of any neurodevelopmental disorder did not yield significant associations between maternal IL-6 levels in pregnancy week 24. Excluding children with neurodevelopmental diagnoses (N=472), yielded β[95%CI]=0.99 [−0.51–2.50], p=0.20. Analyzing only children with neurodevelopmental disorders (N=80), resulting in β[95%CI]=−0.45 [−6.46–5.55], p=0.88.

There were no significant associations to any of the three BRIEF-2 index scores in any of the groups (p>0.17).

Further analysis stratified by sex and according to absence or presence of any neurodevelopmental disorder, can be found in Supplementary Table S5.

The remaining interaction analyses are provided in Supplementary Table S10.

### Sensitivity analyses

#### Associations between BRIEF-2 and EF-PCA

The verbal working memory component was weakly but significantly correlated with most BRIEF-2 subscales. Only a few correlations were observed between the Spatial working memory, respectively, Planning component, and BRIEF-2 subscales (Supplementary Table S8).

#### Maternal IL-6 at 24 weeks pregnancy and estimated FSIQ quotient at age 10

The mean estimated FSIQ for all children was 102.7 (SD=12.1), and slightly higher for girls compared to boys (girls, mean (SD) =104.4(11.3), boys, mean (SD)=101.1(12.7), p<0.001). There was no association between estimated FSIQ and maternal IL-6 during pregnancy (p=0.29). Furthermore, we found no interaction with sex and no significant associations with IL-6 in sex stratified analyses (p-values>0.32).

#### Maternal IL-6 at 24 weeks pregnancy and EF outcomes without PRS adjustment

Analyses without PRS adjustment showed largely consistent results for all significant main and explorative effect modification analyses (see Supplementary Table S9).

### Supplementary analyses

#### Maternal inflammatory markers and SDQ outcomes at age 10

There were significant associations between IL-6 and hs-CRP, respectively, and SDQ outcomes: IL-6 and SDQ total difficulties: Adjusted β[95%CI], p-value: 0.57 [0.168, 0.977], 0.006, IL-6 and SDQ externalizing: 0.36 [0.094, 0.618], 0.008, hs-CRP and SDQ total difficulties score: 0.29 [0.020, 0.558], 0.036 hs-CRP and SDQ externalizing 0.23 [0.054, 0.402], 0.011, (full results in Supplementary Table S11).

#### BRIEF-2 and SDQ outcomes at age 10

BRIEF-2 GEC and SDQ outcomes were highly correlated (Supplementary Table S12).

#### Contributing factors to maternal IL-6 levels during pregnancy

In our covariate association analysis, evaluating contributing factors to IL-6, we found that maternal pre-pregnancy BMI was highly associated with maternal IL-6 levels (unadjusted p<0.001) (Supplementary Table S13). Likewise, in our supplementary analysis, we noted a similar strong association between maternal pre-pregnancy BMI and hs-CRP levels (unadjusted p<0.001).

## Discussion

This study explores thoroughly adjusted analyses of associations between maternal inflammation during pregnancy and EF in middle childhood.

Our main analysis showed a significant association between higher maternal IL-6 levels in pregnancy week 24 and less efficient every day, but not performance-based EF in offspring at the age of 10 years. Specifically, a doubling in maternal IL-6 level (due to the log2 transformation) was associated with a 2.48 points higher score (β[95%CI]=2.48 [0.80- 4.16], p=0.004), on the BRIEF-2 Global Executive Composite score (GEC). A higher score was observed across all three index scores, meaning more problems and thus less efficient EF evident in multiple domains: Behavior Regulation Index, Emotion Regulation Index, and Cognitive Regulation Index.

In our exploratory analysis, when stratifying the analyses based on sex, we found that these associations were solely present in boys, suggesting that only male offspring are susceptible to high levels of IL-6 during pregnancy.

Additionally, stratifying the analysis based on whether or not children had an NDD did not reveal a significant association between IL-6 and EF in children.

A subsequent subanalysis (data not shown) found that the association remained evident for two indices only, namely the GEC and the Behavioral Regulation Index among boys without an NDD. However, this association was not observed among boys with an NDD, possibly due to the limited sample size (N=59). This sex-specific sensitivity to IL-6 inflammation has been previously observed in a recent study where elevated IL-6 levels during pregnancy were associated with the severity of depressive symptoms in male offspring but not in female offspring (Dahabreh & Bibbins-Domingo, 2024). These data could suggest that the fetal developing brain, particularly the male brain, appears to be vulnerable to an adverse intrauterine environment (Kaczkurkin, Raznahan, & Satterthwaite, 2019; McCarthy & Arnold, 2011). One potential explanation might be that male brains develop at a slower pace and thereby are more vulnerable to environmental exposures (Bale, 2016).

Overall, our results are in line with prior findings from another cohort that also found elevated IL-6 levels in pregnancy were associated with diminished EF efficiency (Graham et al., 2018; Rudolph et al., 2018). We did not find an association between the child’s 6 months IL-6 levels and EF at age 10, nor estimated FSIQ, indicating that IL-6 in pregnancy might be adverse specifically for EF.

Our secondary analyses showed no association between elevated maternal hs-CRP and any EF outcomes after covariate adjustment.

Our supplementary analysis explored the relationship between inflammatory markers, BRIEF-2 outcomes, and the SDQ to better understand how behavioral problems intersect with executive function impairments and inflammatory markers.

The BRIEF-2 measures EF in real-life contexts by capturing behavioral indicators of impairment. The BRIEF-2 GEC substantially overlaps with behavioral problems assessed by the Strengths and Difficulties Questionnaire (SDQ), as greater EF impairments on the BRIEF-2 are associated with more severe behavioral problems on the SDQ, underscoring their close relationship (Ng et al., 2024).

We found that IL-6 and hs-CRP were significantly associated with both SDQ outcomes (externalizing subscale and total difficulties score), supporting their role in neurodevelopmental processes and behavioral dysregulation (Allswede et al., 2020; Gustafsson et al., 2020; Hsiao & Patterson, 2011; Morgan et al., 2020; Rasmussen et al., 2019; Smith et al., 2007; Wu et al., 2017). The findings indicate that these inflammatory markers are associated with both behavioral problems and everyday EF, with a somewhat stronger association with EF.

To examine the specificity of BRIEF-2 outcomes, we conducted a bivariate correlation analysis, which revealed strong associations between the BRIEF-2 GEC and both SDQ outcomes. This suggests that the BRIEF-2 captures EF aspects that substantially overlap with broader behavioral problems. The shared variance highlights their close relationship, though these associations may be inflated due to overlapping item content (Ng et al., 2024). Additionally, these associations may have been inflated by shared method variance, as both measures were parent-reported. These limitations should be considered when interpreting the results.

### Cognitive heterogeneity in findings for performance-based EF test scores and BRIEF-2 ratings

Our primary associations were predominantly evident in the outcomes derived from parental-rated BRIEF-2. Although we initially identified an association between maternal IL-6 levels during pregnancy week 24 and the Verbal Working Memory component of the EF-PCA, this association became non-significant after adjusting for covariates. Furthermore, we observed no associations with IL-6 inflammation for the remaining neuropsychological outcomes (Spatial Working memory, respectively, Planning component). We predominantly found non-significant or very weak correlations in our correlation analysis between the lab-based tests and BRIEF-2. This difference in the results aligns with prior research, from across various clinical groups, including individuals with ADHD, emphasizing that performance-based tests of EF and behavioral ratings of EF reflect distinct underlying mental constructs exhibiting minimal overlap, and therefore, they should not be considered equivalent (Toplak et al., 2013; Toplak, Bucciarelli, Jain, & Tannock, 2009). Recent studies in adults with schizophrenia spectrum disorders, as well as adolescents and adults with schizophrenia, also confirm these findings (Haugen et al., 2021; van Aken et al., 2023). Performance-based measures of EF offer valuable insights into a child’s performance in controlled and structured settings with predetermined testing goals and outcomes and estimate processing efficacy and specific cognitive sub-domains. However, performance-based measures may lack ecological validity when it comes to assessing real-world executive functioning and explain little unique variance, whereas rating scales such as BRIEF-2 can be considered more sensitive, though possibly less specific, offer insight into an individual’s attainment of goals in their familiar environment (Toplak et al., 2013). In conclusion, these two different assessment tools were not robustly correlated in our analysis, emphasizing the diversity between performance-based testing and every day rating scale of executive functioning.

The present findings should be considered within the strengths and limitations of the study. The study has several strengths, including a comparably large group of participants from the general population, ensuring its representation. Due to the comprehensive data collection of the COPSAC database, we were able to adjust for multiple relevant covariates, aiding in the control and assessment of potential confounding factors. The included covariates also provided insight into potential factors contributing to elevated inflammation, such as a high pre-pregnancy BMI or preeclampsia.

Certain limitations should be acknowledged. Firstly, our reliance on a single blood measurement during pregnancy, as opposed to a repeated assessment in each trimester, is a limitation. Secondly, our study predominantly focuses on IL-6 as the primary marker of inflammation during pregnancy. Although we acknowledge IL-6 as an important marker of inflammation during pregnancy, other inflammatory markers may also show an association with EF and future research should consider exploring other biomarkers. In our secondary analyses, we observed no association between elevated maternal hs-CRP and any EF outcomes after adjustment.

Furthermore, the absence of direct cognitive measurements for mothers is another limitation, as EF is highly heritable. Instead, EF-PRS was a proxy measure for maternal executive functioning. However, EF-PRS does not accurately represent the actual heritability of EF, making it a sub-optimal measurement due to the low explained variance. Therefore, relying on EF-PRS as a measure could be considered inadequate for drawing firm conclusions regarding the potential genetic contribution. Finally, the inability to calculate the ICC for maternal inflammatory markers is a limitation, as it prevents assessing measurement reliability.

Maternal inflammation can have various origins, including infections, conditions such as preeclampsia, chronic low-grade inflammation linked to obesity, and anticipated higher levels of inflammatory markers during healthy pregnancies (Fu, Tang, Hu, Xiang, & Hu, 2020; Gustafsson et al., 2020; Redman & Sargent, 2009). In our analysis of covariates associated with maternal IL-6 (and hs-CRP), we observed a significant association between maternal pre-pregnancy BMI and IL-6 levels, emphasizing that low-grade inflammation, rather than infections, stands as a potential risk factor for less efficient executive functioning.

However, other mechanisms underlying maternal inflammation during pregnancy and associations with fetal neurodevelopment may be at play. Maternal-fetal IL-6 transfer may be speculated to result in sustained microglia activation that plays a crucial role in brain connectivity and increases the risk of later psychiatric disorders (Kern et al., 2015; Zaretsky, Alexander, Byrd, & Bawdon, 2004).

## Conclusion

Our findings suggest that prenatal inflammation as measured by IL-6 is associated with less efficient every day, but not performance-based EF in offspring at age 10. These findings thereby encourage future studies to further interrogate the impact of early-life inflammation on cognition across diverse populations. If replicated, preventive strategies targeting inflammation in pregnancy may alleviate adverse cognitive outcomes in offspring.

## Supporting information

Mohammadzadeh et al. supplementary material Mohammadzadeh et al. supplementary material

## Abbreviations

BMI: Body Mass Index
BRIEF-2: Behavior Rating Inventory of Executive Function, Second Edition
COPSAC: Copenhagen Prospective Studies on Asthma in Childhood
COPSYCH: COpenhagen Prospective Study on Neuro-PSYCHiatric Development
DSM-5: Diagnostic and Statistical Manual of Mental Disorders, Fifth Edition
EF: Executive Functioning
EF-PCA: Executive Functioning Principal Component Analysis
EF-PRS: Executive Functioning Polygenic Risk Score
FSIQ: full-scale intelligence quotient
HS-CRP: High Sensitivity C-Reactive Protein
ICD-10: International Classification of Diseases, Tenth Revision
IL-6: Interleukin 6
PRS: Polygenic Risk Score

## Supplementary material

The supplementary material for this article can be found at http://doi.org/10.1017/S0033291725000674.
