# Supplementary material for: Maternal interleukin 6 in pregnancy is associated with everyday, but not test-based executive functioning in 10-year-old children
Source: Psychol Med. 2025 Apr 11;55:e112. doi: 10.1017/S0033291725000674 (PMC12094633; doi:10.1017/S0033291725000674)
Supplement: Mohammadzadeh et al. supplementary material [file S0033291725000674sup001.docx]

**SUPPLEMENTARY MATERIALS**

**Methods**

**Polygenic risk score for EF**

Genotyping data in COPSAC2010 cohort was done previously (Ahluwalia et al., 2020) We examined the genetic contributions to the main outcomes by generating maternal PRS for EF. These scores were based on genome-wide association studies conducted with individuals from the UK Biobank (Demontis et al., 2023; Hatoum et al., 2023; Murray et al., 2021). We utilised the Polygenic Risk Score Continuous Shrinkage (PRS-CS) software package to construct the scores, which employs a high-dimensional Bayesian regression framework. This method employs a shrinkage prior to regularise single nucleotide polymorphism (SNP) effects and utilises a linkage disequilibrium (LD) reference panel derived from European samples in the 1000 Genomes Project (Grove et al., 2019). The polygenicity parameter (phi) was automatically estimated. After adjusting SNP effects with PRS-CS, we aggregated SNP effects into PRSs for each individual using PLINK2 software. Subsequently, the scores were standardised to have a mean of 0 and a standard deviation of 1 for each phenotype, thereby regularising SNP effects using a continuous shrinkage prior.

**The Strengths and Difficulties Questionnaire (SDQ)**

The Strengths and Difficulties Questionnaire (SDQ) is a behavioral screening tool designed to assess psychosocial difficulties in children and adolescents. It consists of 25 items divided into five subscales: emotional symptoms, conduct/behavioral problems, hyperactivity/inattention, peer relationship problems, and prosocial behavior. Each item is scored on a 3-point Likert scale, with higher scores indicating greater difficulties (or strengths for the prosocial subscale). The SDQ has been validated across various populations and demonstrates good test-retest reliability, and inter-rater agreement and is commonly used in both research and clinical settings (Goodman, Lamping, & Ploubidis, 2010; Stone, Otten, Engels, Vermulst, & Janssens, 2010). Our analysis focused on broader externalizing behaviors by combining the hyperactivity and behavioral subscales and the total difficulties score (Goodman et al., 2010).

**Results**

**Baseline IL-6, hs-CRP and BRIEF-2 data analyses**

The raw IL-6 data was expectedly skewed, so we performed a log2 transformation (common practice for biomarker concentrations). Following this transformation, the distribution was approximately normal (Kolmogorov-Smirnov test (p-value = 0.08) and thus all IL-6 values were retained). The median raw maternal concentration of IL-6 was 0.30 pg/mL [IQR=0.20-0.42], which is within the normal range for pregnant women (Fu, Tang, Hu, Xiang, & Hu, 2020). There were no significant differences in maternal IL-6 levels in pregnancy between female and male offspring (p=0.14). The median BRIEF-2 Global Executive Composite raw score was 81 [IQR]=[70-97].

The median raw maternal level of hs-CRP was 5.36mg/L [IQR] = [2.62 mg/L – 10.64 mg/L]. There were no significant differences in maternal hs-CRP levels in pregnancy between female and male offspring (p=0.87).

**Supplementary figures and tables**

**Figure S1 Consort diagram**

**
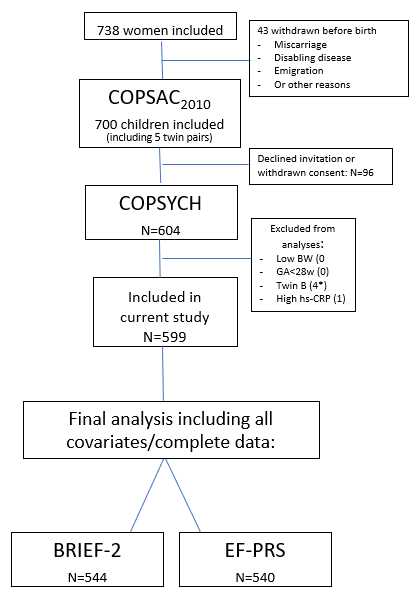
**

*Figure S1 - Consort diagram.*

**A total of 5 twins are in the COPSAC2010 cohort. Thus, the final cohort consisted of 695 mothers and 700 children. One twin B did not participate in the COPSYCH study, therefore only 4 were removed.*

*Abbreviations: BW: birthweight: COPSAC: Copenhagen Prospective Studies on Asthma in Childhood, COPSYCH: COpenhagen Prospective Study on Neuro-PSYCHiatric Development, GA; gestational age, BRIEF-2: Behavior Rating Inventory of Executive Function, Second Edition, EF-PCA: Executive Functioning Principal Component Analysis*

**Figure S2 Biplot Executive Functioning Principal Component Analysis
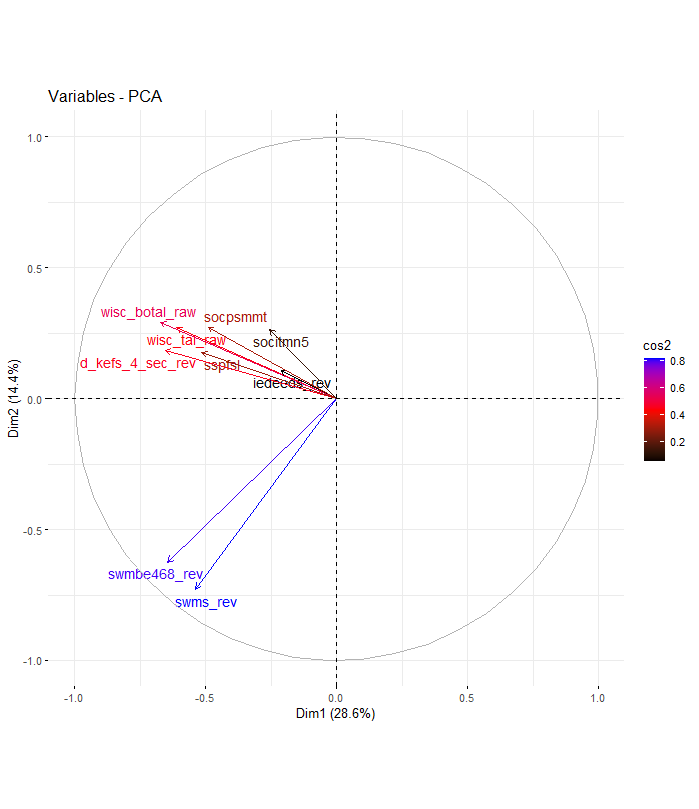
**

*Figure S2 - Biplot, showing the outcome from the Principal Component Analysis.*

*Abbreviations: wisc_tal_raw: Digit Span, Total number of correct forward and backward digit sequencing, (WISC-IV). wisc_botal­_raw: Letter-Number sequencing, Total number of letter-number sequencing, (WISC-IV). sspfsl: Spatial span task, forward span length (CANTAB). socpmmt: One touch stockings of Cambridge, Problems solved in minimum moves, (CANTAB). socitmn5: One touch stockings of Cambridge, Initial Thinking Time Mean (5 Moves), (CANTAB). swmss_rev: Spatial working memory, strategy formation (CANTAB). swmbe468_rev: Spatial working memory, between errors (CANTAB). d_kefs_4_sec_rev: Trail making test 4 - number–letter switching, time to complete in seconds (D-KEFS). iedeeds_rev: Intra-Extra dimensional set shift, extra-dimensional stage errors (CANTAB).*

*Loadings: Principal Component 1 (Verbal Working Memory): d_kefs_4_sec_rev, wisc_tal_raw, wisc_botal_raw, Spatial span task, sspfsl, iedeeds_rev. Principal Component 2 (Spatial Working Memory): socpmmt, socitmn5. Principal Component 3 (Planning): swmbe468_rev, swmss_rev.*

**Table S1 Drop out table**

|  | **COPSYCH participant** | **COPSYCH -**  **Non-participants** | **p-value** | **Range** |
| --- | --- | --- | --- | --- |
| N | 604 | 96 |  |  |
| **Child characteristics** | | | | |
| Sex (males): N (%) | 313 (51.8) | 47 (49.0) | 0.47 |  |
| Ethnicity (non-white): N (%) | 27 (4.5) | 3 (3.1) | 0.51 |  |
| Delivery/birth | | | | |
| Gestational age (days): median [IQR] | 281 [274, 287] | 278 [271, 287] | 0.17 | [206-296] |
| Caesarean section: N (%) | 126 (20.9) | 25 (26.0) | 0.22 |  |
| Season of birth: N (%) |  |  | 0.35 |  |
| Winter | 187 (31.0) | 28 (29.2) |  |  |
| Spring | 166 (27.5) | 20 (20.8) |  |  |
| Summer | 127 (21.0) | 22 (22.9) |  |  |
| Autumn | 124 (20.5) | 26 (27.1) |  |  |
| Birth weight (kg): median [IQR] | 3.56 [3.2, 3.9] | 3.57 [3.18, 3.91] | 0.86 | [1.3-5.2] |
| **Maternal characteristics** | | | | |
| Age at birth: mean (SD) | 32.36 (4.33) | 31.78 (4.5) | 0.15 | [19.1-48.3] |
| Highest education at birth |  | | 0.05 |  |
| - University: N (%) | 174 (28.8) | 20 (20.8) |  |  |
| - High school: N (%) | 336 (55.6) | 66 (68.8) |  |  |
| Smoking (weekly (>1 unit) during pregnancy): N (%) | 44 (7.3) | 10 (10.4) | 0.39 |  |
| Alcohol (weekly (>1 unit) during pregnancy: N (%) | 92 (15.3) | 9 (9.5) | 0.18 |  |
| Preeclampsia: N (%) | 27 (4.5) | 4 (4.2) | 1.00 |  |
| Gestational diabetes: N (%) | 15 (2.5) | 1 (1.0) | 0.61 |  |
| Intervention Vitamin D: N (%) | 252 (49.5) | 46 (59.0) | 0.15 |  |
| Intervention n-3 LCPUFA: N (%) | 307 (50.7) | 42 (43.2) | 0.14 |  |
| **Paternal characteristics** | | | | |
| Age at birth: mean (SD) | 34.61 (5.22) | 33.83 (5.6) | 0.13 | [21.0-53.2] |
| Highest education at birth |  | | 0.19 |  |
| - University: N (%) | 168 (28.5) | 23 (25.0) |  |  |
| - High school: N (%) | 241 (40.8) | 32 (34.8) |  |  |
| Other |  | | | |
| Household income (baseline, per quarter, DKK) |  | | 0.20 |  |
| - <100 000 | 55 (9.1) | 12 (12.5) |  |  |
| - 100 000 - 150 000 | 139 (23.1) | 31 (32.3) |  |  |
| - 150 000 - 200 000 | 178 (29.5) | 22 (22.9) |  |  |
| - 200 000 - 250 000 | 139 (23.1) | 18 (18.8) |  |  |
| - >250 000 | 92 (15.3) | 13 (13.5) |  |  |

**Table S2 Maternal IL-6 pregnancy week 24 and child BRIEF-2 global and indices scores at age 10, Linear regression analysis**

| **Maternal IL-6 pregnancy week 24 and child BRIEF-2 at age 10** | | | | | | | |
| --- | --- | --- | --- | --- | --- | --- | --- |
|  | **N** | **Unadjusted β[95%CI]** | **p-value** | **N** | **Adjusted β[95%CI]** | **p-value** | **FDR adjusted p-value*** |
| **Global Executive Composite** | 577 | 2.62 [1.03,4.21] | 0.001 | 544 | 2.48 [0.80, 4.16] | 0.004 | 0.0125 |
| **Behavior Regulation Index** | 577 | 0.54 [0.20, 0.87] | 0.002 | 544 | 0.52 [0.15-0.88] | 0.006 | 0.025 |
| **Emotion Regulation Index** | 577 | 0.56 [0.07,1.06] | 0.03 | 544 | 0.58 [0.03-1.12] | 0.038 | 0.05 |
| **Cognitive Regulation Index** | 577 | 1.53 [0.60, 2.48] | 0.002 | 544 | 1.39 [0.40-2.38] | 0.007 | 0.0375 |

| **Maternal IL-6 pregnancy week 24 and child BRIEF-2 at age 10 - sex stratified** | | | | | | |
| --- | --- | --- | --- | --- | --- | --- |
| **Female** | | | | | | |
|  | **N** | **Unadjusted β[95%CI]** | **p-value** | **N** | **Adjusted β[95%CI]** | **p-value** |
| **Global Executive Composite** | 283 | 1.32 [-0.58, 3.21] | 0.18 | 267 | 1.08 [-0.91, 3.06] | 0.29 |
| **Behavior Regulation Index** | 283 | 0.22 [-0.15, 0.59] | 0.25 | 267 | 0.15 [-0.26, 0.56] | 0.47 |
| **Emotion Regulation Index** | 283 | 0.23 [-0.37, 0.84] | 0.45 | 267 | 0.18 [-0.47, 0.84] | 0.59 |
| **Cognitive Regulation Index** | 283 | 0.87 [-0.26, 1.99] | 0.13 | 267 | 0.74 [-0.40, 1.89] | 0.21 |
| **Male** | | | | | | |
|  | **N** | **Unadjusted β[95%CI]** | **p-value** | **N** | **Adjusted β[95%CI]** | **p-value** |
| **Global Executive Composite** | 294 | 4.97 [2.23, 7.71] | <0.001 | 277 | 5.28 [2.19, 8.37] | <0.001 |
| **Behavior Regulation Index** | 294 | 1.10 [0.47, 1.72] | <0.001 | 277 | 1.23 [0.52, 1.93] | <0.001 |
| **Emotion Regulation Index** | 294 | 1.15 [0.40, 2.37] | 0.007 | 277 | 1.38 [0.40, 2.37] | 0.006 |
| **Cognitive Regulation Index** | 294 | 2.72 [1.07, 4.36] | 0.001 | 277 | 2.67 [0.80, 4.54] | 0.005 |

*****P-values adjusted with Benjamini-Hochberg correction method

**Table S3 Maternal hs-CRP pregnancy week 24 and child BRIEF-2 global and indices scores at age 10, linear regression analysis**

| **Maternal CRP pregnancy week 24 and child BRIEF-2 at age 10** | | | | | | |
| --- | --- | --- | --- | --- | --- | --- |
|  | **N** | **Unadjusted β[95%CI]** | **p-value** | **N** | **Adjusted β[95%CI]** | **p-value** |
| **Global Executive Composite** | 577 | 1.57 [0.50, 2.64] | 0.004 | 556 | 0.90 [-0.28, 2.00] | 0.14 |
| **Behaviour Regulation Index** | 577 | 0.26 [0.03, 0.48] | 0.03 | 556 | 0.11 [-0.14, 0.35] | 0.40 |
| **Emotion Regulation Index** | 577 | 0.30 [-0.03, 0.64] | 0.08 | 556 | 0.15 [-0.22, 0.51] | 0.44 |
| **Cognitive Regulation Index** | 577 | 1.01 [0.37, 1.65] | 0.002 | 556 | 0.61 [-0.07, 1.29] | 0.08 |

**Table S4 Maternal hs-CRP and child EF test outcomes**

| **Linear regression analysis, hs-CRP and EF test outcomes** | | | | | | | |
| --- | --- | --- | --- | --- | --- | --- | --- |
| **Neurocognitive subdomain** | **Outcome variable** | **N** | **Unadjusted β[95%CI]** | **p-value** | **N** | **Adjusted β[95%CI]** | **p-value** |
| Intra-Extra dimensional set shift (CANTAB) | -Extra-dimensional stage errors | 582 | -0.07 [-0.61, 0.46] | 0.79 | 553 | -0.05 [-0.61, 0.52] | 0.87 |
| Trail making test 4 - number–letter switching (D-KEFS) | -Time to complete in seconds | 583 | 0.73 [-1.27, 2.73] | 0.48 | 553 | 0.55 [-1.52, 2.62] | 0.60 |
|  |  |  |  |  |  |  |  |
| Spatial span task (CANTAB) | - Forward span length | 585 | 0.02 [-0.04, 0.09] | 0.46 | 555 | 0.03 [-0.04, 0.09] | 0.44 |
| Spatial working memory (CANTAB) | -Between errors | 585 | 0.14 [-0.25, 0.53] | 0.47 | 555 | 0.04 [-0.36,0.45] | 0.84 |
|  | -Strategy formation | 585 | 0.018 [-0.09, 0.12] | 0.74 | 555 | -0.02 [-0.13, 0.09] | 0.73 |
| One touch stockings of Cambridge (CANTAB) | -Problems solved in minimum moves | 584 | -0.02 [-0.12, 0.08] | 0.64 | 554 | -0.01 [ -0.11, 0.10] | 0.86 |
|  | -Mean Initial Thinking Time (5 Moves) | 580 | 22.2 [-210, 255] | 0.85 | 550 | -16.8 [ -266, 232] | 0.90 |
| Digit Span (WISC-IV) | -Total number of correct forward and backward digit sequencing | 584 | -0.14 [-0.28, -0.01] | 0.04 | 555 | -0.11 [-0.25, 0.02] | 0.10 |
| Letter-Number sequencing (WISC-IV) | -Total number of letter-number sequencing | 582 | -0.16 [-0.31, -0.01] | 0.04 | 553 | -0.09 [-0.24, 0.07] | 0.28 |

**Table S5 Analyses stratified by both sex and status of neurodevelopmental disorder, linear regression analysis**

| **Male without any NDD** | | | | | | |
| --- | --- | --- | --- | --- | --- | --- |
| **Variable** | **Unadjusted β[95%CI]** | **p-value** | **N** | **Adjusted β[95%CI]** | **p-value** | **N** |
| Global Executive Composite | 4.44 [ 1.92, 6.96] | <0.001 | 233 | 2.85 [0.09, 5.61] | <0.05 | 224 |
| Behavior Regulation Index | 1.04 [0.47, 1.61] | <0.001 | 233 | 0.76 [0.14, 1.38] | <0.05 | 224 |
| Emotion Regulation Index | 1.10 [0.24, 1.76] | <0.05 | 233 | 0.70 [-0.16, 1.55] | 0.11 | 224 |
| Cognitive Regulation Index | 2.40 [0.79, 4.00] | <0.05 | 233 | 1.40 [-0.37, 3.17] | 0.12 | 224 |
| **Male with any NDD** | | | | | | |
| **Variable** | **Unadjusted β[95%CI]** | **p-value** | **N** | **Adjusted β[95%CI]** | **p-value** | **N** |
| Global Executive Composite | 0.18 [-5.22, 5.59] | 0.95 | 60 | 4.52 [ -2.31, 11.3] | 0.20 | 59 |
| Behavior Regulation Index | -0.02 [ -1.35, 1.35] | 1.00 | 60 | 0.93 [-0.88, 2.74] | 0.32 | 59 |
| Emotion Regulation Index | -0.13 [-2.07, 1.82] | 0.90 | 60 | 0.65 [-0.91, 4.21] | 0.21 | 59 |
| Cognitive Regulation Index | 0.31 [-3.07, 3.69] | 0.86 | 60 | 1.94 [-2.62, 6.49] | 0.41 | 59 |
| **Female without any NDD** | | | | | | |
| **Variable** | **Unadjusted β[95%CI]** | **p-value** | **N** | **Adjusted β[95%CI]** | **p-value** | **N** |
| Global Executive Composite | 0.62 [ -1.10, 2.34] | 0.48 | 258 | 0.03 [-1.82, 1.88] | 0.97 | 248 |
| Behavior Regulation Index | 0.03 [ -0.31, 0.37] | 0.85 | 258 | -0.09 [-0.46, 0.28] | 0.63 | 248 |
| Emotion Regulation Index | 0.02 [-0.55, 0.59] | 0.94 | 258 | -0.09 [ -0.72, 0.54] | 0.79 | 248 |
| Cognitive Regulation Index | 0.57 [-0.46, 1.59] | 0.28 | 258 | 0.21 [-0.88, 1.30] | 0.71 | 248 |
| **Female with any NDD** | | | | | | |
| **Variable** | **Unadjusted β[95%CI]** | **p-value** | **N** | **Adjusted β[95%CI]** | **p-value** | **N** |
| Global Executive Composite | -3.22 [-11.6, 5.20] | 0.46 | 25 | -18.5 [-33.6, -3.34] | 0.97 | 24 |
| Behavior Regulation Index | 0.007 [ -1.78, 1.79] | 0.99 | 25 | -3.26 [-7.20, 0.68] | 0.18 | 24 |
| Emotion Regulation Index | -0.64 [-3.41, 2.13] | 0.66 | 25 | -5.08 [-9.55, -0.61] | 0.09 | 24 |
| Cognitive Regulation Index | -2.59 [ -7.73, 2.54] | 0.33 | 25 | -10.1 [-20.7, 0.45] | 0.13 | 24 |

**Table S6 Maternal IL-6 pregnancy week 24 and child EF-PCA at age 10, linear regression analysis**

| **Maternal IL-6 pregnancy week 24 and child EF-PCA at age 10** | | | | | | |
| --- | --- | --- | --- | --- | --- | --- |
|  | **N** | **Unadjusted β[95%CI]** | **p-value** | **N** | **Adjusted β[95%CI]** | **p-value** |
| **Verbal WM** | 571 | -0.16 [-0.29, -0.04] | 0.01 | 540 | -0.09 [-0.22, 0.037] | 0.17 |
| **Spatial WM** | 571 | -0.03 [-0.12, 0.06] | 0.57 | 540 | -0.01 [-0.11, 0.09] | 0.77 |
| **Planning** | 571 | 0.01 [-0.08, 0.09] | 0.87 | 540 | -0.007 [-0.10, 0.09] | 0.88 |

**Table S7 Maternal IL-6 and child EF test outcomes**

| **Linear regression analysis, IL-6 and EF test outcomes** | | | | | | | |
| --- | --- | --- | --- | --- | --- | --- | --- |
| **Neurocognitive subdomain** | **Outcome variable** | **N** | **Unadjusted β[95%CI]** | **p-value** | **N** | **Adjusted β[95%CI]** | **p-value** |
| Intra-Extra dimensional set shift (CANTAB) | -Extra-dimensional stage errors | 582 | 0.22 [-0.58, 1.02] | 0.59 | 553 | 0.07 [-0.78, 0.92] | 0.87 |
| Trail making test 4 - number–letter switching (D-KEFS) | -Time to complete in seconds | 583 | 3.18 [0.21, 6.15] | 0.04 | 553 | 2.78 [-0.33, 5.90] | 0.08 |
|  |  |  |  |  |  |  |  |
| Spatial span task (CANTAB) | - Forward span length | 585 | -0.04 [-0.13, 0.06] | 0.47 | 555 | -0.04 [-0.14, 0.06] | 0.40 |
| Spatial working memory (CANTAB) | -Between errors | 585 | 0.39 [-0.19, 0.97] | 0.19 | 555 | 0.14 [-0.47, 0.75] | 0.65 |
|  | -Strategy formation | 585 | 0.09 [-0.06, 0.24] | 0.25 | 555 | 0.04 [-0.12 0.21] | 0.61 |
| One touch stockings of Cambridge (CANTAB) | -Problems solved in minimum moves | 584 | -0.03 [-0.18, 0.12] | 0.66 | 554 | -0.01 [-0.16, 0.15] | 0.94 |
|  | -Mean Initial Thinking Time (5 Moves) | 580 | -237 [-581. 107] | 0.18 | 550 | -321 [-69, 50.5] | 0.09 |
| Digit Span (WISC-IV) | -Total number of correct forward and backward digit sequencing | 584 | -0.25 [-0.44, -0.05] | 0.01 | 555 | -0.21 [-0.41, -0.01] | 0.05* |
| Letter-Number sequencing (WISC-IV) | -Total number of letter-number sequencing | 582 | -0.27 [-0.49, -0.04] | 0.02 | 553 | -0.17 [-0.40, 0.06] | 0.14 |

* Observed p-value: 0.0499

**Table S8 Overview of interactions**

Interaction between maternal IL-6 and covariates in relation to Global Executive Composite score from BRIEF-2, in models adjusted for remaining covariates:

|  | **Interaction P-value** |
| --- | --- |
| Sex=male | 0.01* |
| Maternal education | 0.36 |
| Household income (DKK, per quarter baseline) | 0.13 |
| Maternal pre-pregnancy BMI | 0.22 |
| Preeclampsia | 0.93 |
| Birth weight | 0.77 |
| Child PRS for EF | 0.58 |
| Neurodevelopmental disorder (Y/N) | 0.94 |

*: p<0.05. reference level

**Table S9 Correlation analyses (Spearman's rank), between corresponding Behavior Rating Inventory of Executive Function subscales scores, Second Edition and EF-PCA component scores**

| **Component from EF-PCA** | **Subscale BRIEF-2** | **Estimate, p-value** |
| --- | --- | --- |
| Verbal working memory | Inhibit | -0.20, p<0.001* |
|  | Self-Monitor | -0.21, p<0.001* |
|  | Shift | -0.17, p<0.001* |
|  | Emotional Control | -0.11, p=0.007* |
|  | Initiate/Task Completion | -0.27, p<0.001* |
|  | Working Memory | -0.35, p<0.001* |
|  | Plan/Organize | -0.29, p<0.001* |
|  | Task-Monitor | -0.30, p<0.001* |
|  | Organization of Materials | -0.21, p<0.001* |
| Spatial working memory | Inhibit | -0.07, p=0.08 |
|  | Self-Monitor | -0.08, p=0.048* |
|  | Shift | -0.06, p=0.16 |
|  | Emotional Control | -0.09, p=0.03* |
|  | Initiate/Task Completion | 0.04, p=0.33 |
|  | Working Memory | -0.05, p=0.24 |
|  | Plan/Organize | -0.04, p=0.29 |
|  | Task-Monitor | -0.07, p=0.08 |
|  | Organization of Materials | -0.06, p=0.15 |
| Planning | Inhibit | -0.001, p=0.09 |
|  | Self-Monitor | 0.11, p=0.01* |
|  | Shift | 0.05, p=0.24 |
|  | Emotional Control | 0.03, p=0.50 |
|  | Initiate/Task Completion | 0.07, p=0.12 |
|  | Working Memory | 0.07, p=0.10 |
|  | Plan/Organize | 0.04, p=0.31 |
|  | Task-Monitor | 0.005, p=0.90 |
|  | Organization of Materials | 0.04, p=0.31 |

*: p<0.05. reference level

**Table S10 Maternal IL-6 at 24 weeks pregnancy and EF outcomes without PRS adjustment, linear regression analysis**

| **Maternal IL-6 pregnancy week 24 and child BRIEF-2 at age 10 – WITHOUT PRS as a covariate** | | | |
| --- | --- | --- | --- |
|  | **N** | **Adjusted β[95%CI]** | **p-value** |
| **Global Executive Composite** | 559 | 2.37 [0.70, 4.04] | 0.006 |
| **Behavior Regulation Index** | 599 | 0.53 [0.17, 0.88] | 0.004 |
| **Emotion Regulation Index** | 599 | 0.57 [0.04, 1.11] | 0.037 |
| **Cognitive Regulation Index** | 599 | 1.27 [0.28, 2.27] | 0.012 |
| **Maternal IL-6 pregnancy week 24 and child BRIEF-2 at age 10 - sex stratified – Male – WITHOUT PRS as a covariate** | | | |
|  | **N** | **Adjusted β[95%CI]** | **p-value** |
| **Global Executive Composite** | 284 | 5.13 [2.18, 8.08] | <0.001 |
| **Behavior Regulation Index** | 284 | 1.21 [0.54, 1.88] | <0.001 |
| **Emotion Regulation Index** | 284 | 1.33 [0.40, 2.27] | 0.006 |
| **Cognitive Regulation Index** | 284 | 2.59 [0.80, 4.38] | 0.005 |

**Table S11 Associations between maternal inflammatory markers and SDQ outcomes at age 10 (linear regression analysis)**

| **Maternal IL-6 pregnancy week 24 and child SDQ outcomes at age 10** | | | | | | |
| --- | --- | --- | --- | --- | --- | --- |
|  | **N** | **Unadjusted β[95%CI]** | **p-value** | **N** | **Adjusted β[95%CI]** | **p-value** |
| **SDQ total difficulties score** | 571 | 1.04 [0.217, 1.87] | 0.014 | 540 | 0.57 [0.168, 0.977] | 0.006 |
| **SDQ externalizing subscale** | 571 | 0.38 [0.122, 0.630] | 0.004 | 540 | 0.36 [0.094, 0.618] | 0.008 |
| **Maternal hs-CRP pregnancy week 24 and child SDQ outcomes at age 10** | | | | | | |
|  | **N** | **Unadjusted β[95%CI]** | **p-value** | **N** | **Adjusted β[95%CI]** | **p-value** |
| **SDQ total difficulties score** | 571 | 0.28 [0.108, 0.448] | 0.001 | 540 | 0.29 [0.020, 0.558] | 0.036 |
| **SDQ externalizing subscale** | 571 | 1.04 [ 0.211, 1.87] | 0.014 | 540 | 0.23 [0.054, 0.402] | 0.011 |

**Table S12 BRIEF-2 - Global Executive Composite and SDQ at age 10 (Spearman´s Correlation analysis)**

| **BRIEF – Global Executive Composite score and SDQ outcomes at age 10** | | |
| --- | --- | --- |
|  | **Rho (p-value)** | **Shared variance** |
| **SDQ total difficulties score** | 0.743 (p<0.0001) | 55.2% |
| **SDQ externalizing subscale** | 0.737 (p<0.0001) | 54.3% |

**Table S13 associations analysis between covariates and maternal log2(IL-6) gestational week 24**

| **Covariate** | **β[95%CI]** | **p-value** |
| --- | --- | --- |
| Parity | -0.07 [-0.18, 0.04] | 0.19 |
| Maternal age (years) | 0.01 [-0.01, 0.03] | 0.31 |
| Maternal level of education   - Elementary - College - Tradesman - Medium - University | *Reference level*  -0.35 [-0.94, 0.26]  -0.35 [ -0.87, 0.16]  -0.28 [-0.78, 0.21]  -0.53 [-1.04, -0.03] | 0.36* |
| Antibiotics in pregnancy | 0.09 [-0.09, 0.26] | 0.33 |
| Smoking in pregnancy | 0.30 [-0.02, 0.61] | 0.07 |
| Maternal pre-pregnancy BMI | 0.06 [0.04, 0.07] | <0.001 |
| Paternal age (years) | 0.01 [-0.01, 0.02] | 0.47 |
| Household income (DKK, per quarter, baseline):   - <100 000 - 100 000 - 150 000 - 150 000 - 200 000 - 200 000 - 250 000 - >250 000 | -0.01 [-0.33, 0.32]  *Reference level*  -0.02 [-0.26, 0.20]  -0.31 [-0.55, -0.06]  -0.13 [ -0.40, 0.15] | 0.22* |
| Maternal PRS for EF | 0.02 [-0.06, 0.11] | 0.59 |

Univariate linear regression.

*p-value reflects likelihood ratio test for overall effect of level of household income and maternal education level.

**References**

Ahluwalia, T. S., Eliasen, A. U., Sevelsted, A., Pedersen, C.-E. T., Stokholm, J., Chawes, B., … Bønnelykke, K. (2020). FUT2-ABO epistasis increases the risk of early childhood asthma and Streptococcus pneumoniae respiratory illnesses. *Nature Communications*, *11*(1), 6398. https://doi.org/10.1038/s41467-020-19814-6

Demontis, D., Walters, G. B., Athanasiadis, G., Walters, R., Therrien, K., Nielsen, T. T., … Børglum, A. D. (2023). Genome-wide analyses of ADHD identify 27 risk loci, refine the genetic architecture and implicate several cognitive domains. *Nature Genetics*, *55*(2), 198–208. https://doi.org/10.1038/s41588-022-01285-8

Fu, Y., Tang, L., Hu, M., Xiang, Z., & Hu, Y. (2020). Changes of serum interleukin-6 in healthy pregnant women and establishment of relevant reference intervals. *Clinica Chimica Acta*, *502*, 116–119. https://doi.org/10.1016/j.cca.2019.12.013

Goodman, A., Lamping, D. L., & Ploubidis, G. B. (2010). When to use broader internalising and externalising subscales instead of the hypothesised five subscales on the Strengths and Difficulties Questionnaire (SDQ): Data from British parents, teachers and children. *Journal of Abnormal Child Psychology*, *38*(8), 1179–1191. https://doi.org/10.1007/s10802-010-9434-x

Grove, J., Ripke, S., Als, T. D., Mattheisen, M., Walters, R. K., Won, H., … Børglum, A. D. (2019). Identification of common genetic risk variants for autism spectrum disorder. *Nature Genetics*, *51*(3), 431–444. https://doi.org/10.1038/s41588-019-0344-8

Hatoum, A. S., Morrison, C. L., Mitchell, E. C., Lam, M., Benca-Bachman, C. E., Reineberg, A. E., … Friedman, N. P. (2023). Genome-wide Association Study Shows That Executive Functioning Is Influenced by GABAergic Processes and Is a Neurocognitive Genetic Correlate of Psychiatric Disorders. *Biological Psychiatry*, *93*(1), 59–70. https://doi.org/10.1016/j.biopsych.2022.06.034

Murray, G. K., Lin, T., Austin, J., McGrath, J. J., Hickie, I. B., & Wray, N. R. (2021). Could Polygenic Risk Scores Be Useful in Psychiatry?: A Review. *JAMA Psychiatry*, *78*(2), 210–219. https://doi.org/10.1001/jamapsychiatry.2020.3042

Stone, L. L., Otten, R., Engels, R. C. M. E., Vermulst, A. A., & Janssens, J. M. A. M. (2010). Psychometric properties of the parent and teacher versions of the strengths and difficulties questionnaire for 4- to 12-year-olds: A review. *Clinical Child and Family Psychology Review*, *13*(3), 254–274. https://doi.org/10.1007/s10567-010-0071-2
